# Development of a Nomogram Model for Predicting Mortality Risk in Critically Ill Children with Influenza

**DOI:** 10.3390/children13101293

**Published:** 2026-09-23

**Authors:** Yingying Ye, Zhiling Zhang, Jianlong Chen, Huiming Sun, Zhenjiang Bai, Weifang Zhou, Yanqun Jiang

**Affiliations:** 1 Department of Infectious Diseases, Children’s Hospital of Soochow University, No. 303, Jingde Road, Suzhou 215003, China; yeyingying0918@163.com (Y.Y.); zhangzhiling6128@163.com (Z.Z.); chenjianlong6677@163.com (J.C.); 2 Department of Respiratory Medicine, Children’s Hospital of Soochow University, No. 303, Jingde Road, Suzhou 215003, China; shm_1985@126.com; 3 Department of Pediatric Intensive Care Unit, Children’s Hospital of Soochow University, No. 92, Zhongnan Road, Suzhou 215025, China; bbyshu@hotmail.com

**Keywords:** critically ill children, influenza, mortality, nomogram, predictors

## Abstract

**Highlights:**

**What are the main findings?**

• Five variables, including Hgb, PLT, UREA, ALB, and confusion, were identified as key predictors of mortality in critically ill children with influenza.
• A mortality risk nomogram integrating these five predictors showed good discrimination and calibration after internal validation.

**What are the implications of the main findings?**

• The nomogram provides an individualized estimate of mortality risk using readily available clinical and laboratory variables.
• The model may support early identification of critically ill children at high risk of death and assist clinical risk stratification and decision-making.

**Abstract:**

Background: Influenza virus commonly infects children. Children with critical infection may develop influenza-associated encephalopathy or encephalitis, conditions rarely seen in adults and associated with high mortality. This results in growing healthcare resource consumption and family burden. Objective: This study aimed to identify the predictors for death in critically ill children with influenza, so as to facilitate early recognition of high-risk patients, guide clinical interventions, and reduce mortality. Methods: We retrospectively analyzed the clinical data of critically ill children with influenza admitted to the Children’s Hospital of Soochow University between January 2018 and January 2026. L1-penalized binomial logistic regression (LASSO) was used for variable selection. Variables selected by LASSO were entered into a Firth penalized logistic regression model to identify independent predictors of mortality, based on which a nomogram was constructed to estimate mortality risk. Model discrimination was assessed using the receiver operating characteristic (ROC) curve and area under the curve (AUC). Bootstrap resampling was performed for internal validation and optimism correction. Model calibration was assessed using calibration curves, and decision curve analysis (DCA) was performed to evaluate the clinical net benefit of the model. Results: A total of 113 critically ill children with influenza were included, of whom 34 died, corresponding to a mortality rate of 30.1%. LASSO regression was used for variable selection, followed by Firth penalized logistic regression to develop a mortality risk prediction model. The final model included five variables: Hgb, PLT, UREA, ALB, and confusion. A mortality risk nomogram was developed based on these five predictors and internally validated using bootstrap resampling. The ROC curve showed good discrimination of the nomogram in internal validation. The calibration curve demonstrated good agreement between the predicted probabilities and observed outcomes. DCA showed that the nomogram provided a higher net benefit across a certain range of threshold probabilities, indicating its potential clinical utility. Conclusions: Hgb, PLT, UREA, ALB, and confusion are important predictors of mortality in critically ill children with influenza. The nomogram developed based on these predictors showed good discrimination and calibration, as well as potential clinical net benefit, and may serve as an adjunctive tool for early identification of mortality risk and clinical decision-making in critically ill children with influenza.

## 1. Introduction

Influenza virus infection is a leading cause of respiratory disease in children and imposes a substantial global burden [1]. During seasonal epidemics, approximately 20% to 30% of children are infected each year. Among them, 3 to 5 million develop severe illness, resulting in 290,000 to 650,000 deaths [2,3]. Although most infections are self-limiting, some cases may rapidly progress to multiple organ failure and even death. Their poor prognosis is often closely associated with underlying conditions including asthma, congenital heart disease, and immunodeficiency, as well as various complications [4,5]. Notably, some children may develop acute necrotizing encephalopathy (ANE), a severe form of influenza-associated encephalopathy characterized by rapid neurological deterioration and potentially resulting in permanent neurological sequelae or death [6,7,8].

Influenza vaccination remains an important public health strategy for preventing infection, reducing disease severity, and lowering influenza-related mortality [9]. The World Health Organization (WHO) and current clinical guidelines recommend annual influenza vaccination for children, particularly those at increased risk of severe disease [10]. However, antigenic variation of influenza viruses, insufficient vaccine coverage, and individual differences in immune responses may contribute to severe infection or even death in some children despite preventive measures [11,12,13]. Therefore, in addition to strengthening influenza vaccination strategies, early identification of predictors of mortality in critically ill children with influenza and timely intervention are essential for improving disease management and reducing mortality. In this study, we retrospectively investigated predictors of mortality among critically ill children with influenza and developed a nomogram to estimate mortality risk, providing a basis for early risk stratification and individualized intervention in clinical practice.

## 2. Materials and Methods

### 2.1. Study Population

Children who were hospitalized in the intensive care unit of the Children’s Hospital of Soochow University for critical influenza from January 2018 to January 2026 were enrolled. All included children met the following criteria [14]: (1) Influenza A and/or B virus infection was confirmed by viral isolation, antigen detection, and/or reverse transcription polymerase chain reaction using specimens collected from nasopharyngeal swabs, throat swabs, sputum, pleural effusion, or bronchoalveolar lavage fluid within 7 days before admission to 3 days after admission. (2) Patients had at least one of the following conditions: respiratory failure, ANE, shock, multiple organ dysfunction, or other severe clinical conditions requiring intensive care. (3) Patients were younger than 18 years. (4) Patients with incomplete clinical data or those discharged against medical advice without a clinical outcome available within 28 days after discharge were excluded. A small number of laboratory variables were missing in four patients, mainly involving blood routine indicators, because some of these tests were performed at outpatient clinics of other hospitals and the corresponding results were unavailable in our medical records.

The primary outcome was death. Patients who died during hospitalization were classified as having experienced the outcome event. For patients discharged against medical advice, follow-up was conducted until 28 days after discharge to determine their vital status. Death occurring within 28 days after discharge was considered an outcome event, whereas patients who remained alive at the end of the 28-day follow-up were classified as survivors. Based on clinical outcomes during hospitalization, children were classified into the survival and death groups (Figure 1).

### 2.2. Data Collection

Clinical information was collected from patient records in the electronic medical record system, including general characteristics, clinical features, laboratory parameters, and outcome measures. All laboratory variables used in the analysis were obtained from the first blood samples collected within 24 h of admission to capture the early clinical status of critically ill children with influenza. In addition, commonly used hematological inflammatory markers were calculated from baseline complete blood counts [15]. These included NLR (neutrophil-to-lymphocyte ratio), MLR (monocyte-to-lymphocyte ratio), PLR (platelet-to-lymphocyte ratio), SII (systemic immune inflammation index, defined as platelet count × neutrophil count/lymphocyte count), and SIRI (systemic inflammatory response index, defined as neutrophil count × monocyte count/lymphocyte count).

### 2.3. Statistical Analysis

This study used SPSS 27 and R 4.4.1 for data analysis. The included characteristics comprised both numerical and categorical variables. For data following a normal or approximately normal distribution, results were expressed as mean ± standard deviation ($\bar{x}$ ± s) and compared between groups using the independent samples *t* test. For continuous variables with non-normal distributions, data were presented as the median and interquartile range, M (P25, P75), and compared between groups using the Mann–Whitney U test. Categorical variables were presented as counts and percentages [*n* (%)] and compared using the Pearson chi-square test or Fisher’s exact test when the expected frequencies were small.

Candidate variables were selected based on clinical relevance and evidence from previous studies rather than prescreened according to statistical significance in univariable analyses. L1-penalized LASSO regression was then performed using the glmnet package in R to select variables, with five-fold cross-validation used to determine the optimal regularization parameter (λ). Before LASSO analysis, the predictors were internally standardized using the default standardization procedure of glmnet. Variables with non-zero regression coefficients were retained and subsequently entered into a Firth penalized logistic regression model to develop the final prediction model, based on which a nomogram was constructed.

Model discrimination was assessed using the ROC curve and the AUC. Internal validation was performed using 1000 bootstrap resamples to estimate and correct optimism. For each bootstrap replicate, a sample of the same size as the original dataset was drawn with replacement, and the prediction model was refitted using the bootstrap sample. The fitted model was evaluated on both the bootstrap sample and the original dataset. The difference between the two AUC estimates was defined as the optimism for that bootstrap replicate. The mean optimism across all 1000 bootstrap replicates was then subtracted from the apparent AUC obtained from the original dataset to derive the optimism-corrected AUC. Model calibration was assessed using calibration curves to compare predicted probabilities with observed probabilities. DCA was performed to evaluate the clinical net benefit across a range of threshold probabilities. All statistical results were considered significant at *p* < 0.05.

## 3. Results

### 3.1. Baseline Characteristics of the Study Children

A total of 113 children with critical influenza were included. The median (interquartile range) age was 4.63 (2.42, 7.19) years, with 53.1% (60/113) male and 46.9% (53/113) female. Among them, 79 (69.9%) critically ill patients survived and were assigned to the survival group, while 34 (30.1%) died and were assigned to the non-survival group. There were no statistically significant differences in sex or age between the two groups (*p* > 0.05). Likewise, no significant differences were observed in underlying diseases or preterm birth rates (*p* > 0.05). The results are shown in Table 1.

### 3.2. Clinical and Laboratory Characteristics

Significant differences in clinical and laboratory characteristics were observed between the non-survival and survival groups. Compared with the survival group, the non-survival group had a higher proportion of patients presenting with confusion upon admission (*p* < 0.05). Additionally, the days of fever and length of hospital stay were shorter in the non-survival group than in the survival group (*p* < 0.05), as shown in Table S1. Regarding laboratory findings, except for white blood cells (WBC), neutrophil (NE), c-reactive protein (CRP), triglycerides (TG), total cholesterol (TC), and globulin, most laboratory parameters differed significantly between the two groups (*p* < 0.05), as presented in Table S2. The distributions of selected continuous laboratory variables are further shown as histograms in Figure S1. In terms of complications, the non-survival group showed significantly higher proportions of influenza-associated encephalopathy or encephalitis, respiratory failure, sepsis, coagulation dysfunction, heart failure, kidney injury or renal insufficiency, shock, multiple organ failure, and gastrointestinal bleeding compared with the survival group (*p* < 0.001), as detailed in Table S3.

### 3.3. Feature Selection

To identify potential predictors of mortality in critically ill children with influenza, candidate variables were selected based on clinical relevance and evidence from previous studies rather than prescreened according to statistical significance in univariable analyses. A total of 38 candidate variables were included in the LASSO regression analysis. LASSO logistic regression with an L1 penalty was performed for variable selection, with five-fold cross-validation used to determine the optimal regularization parameter. The cross-validation results showed that the minimum binomial deviance was achieved at an optimal λ of 0.042945 (Figure 2a,b). At this λ value, five variables, namely Hgb, PLT, UREA, ALB, and confusion, had non-zero regression coefficients and were therefore retained for subsequent Firth penalized logistic regression analysis.

### 3.4. Nomogram Construction and Bootstrap Internal Validation

The five candidate predictors selected by LASSO regression were entered into a Firth penalized logistic regression model (Table 2). The final model included Hgb, PLT, UREA, albumin, and confusion. The logit equation was as follows: Logit(P) = −1.994227 + 0.045652 × Hgb − 0.007668 × PLT + 0.344343 × UREA − 0.142271 × albumin + 0.986438 × confusion. A nomogram integrating these five predictors was constructed based on the Firth penalized logistic regression model to provide an intuitive estimate of mortality risk in critically ill children with influenza (Figure 3). For example, a critically ill child with influenza had no confusion at admission, a PLT count of 80 × 10^9^/L, a UREA level of 4.5 mmol/L, an Hgb level of 139 g/L, and an ALB level of 40.2 g/L. The corresponding total score was 214 points, yielding an estimated mortality risk of 0.393.

ROC curve analysis showed that the model achieved an AUC of 0.9356 (95% CI: 0.8941–0.9771). The optimal cutoff value was 0.2184, with a Youden index of 0.7219. At this cutoff, the model achieved a sensitivity of 91.18% (95% CI: 76.32–98.14%) and a specificity of 81.01% (95% CI: 70.62–88.97%) (Figure 4). Internal validation was performed using 1000 bootstrap resamples, all of which yielded valid model estimates. The mean bootstrap optimism was 0.0225 (standard deviation, 0.0244), resulting in an optimism-corrected AUC of 0.9131. The calibration curve remained generally close to the ideal calibration line, with a mean absolute error of 0.058 and a mean squared error of 0.00585. The calibration intercept was 0.003 (95% CI: −0.877–0.858), and the calibration slope was 0.776 (95% CI: 0.408–1.088), indicating good model calibration (Figure 5).

Furthermore, we performed DCA on the nomogram model to compare the net benefit of different clinical decision-making approaches. Figure 6 shows the net benefit across different threshold probabilities. The nomogram showed a positive net benefit across a range of threshold probabilities, suggesting potential clinical utility.

## 4. Discussion

Highly contagious, influenza virus commonly infects children and causes high mortality due to their immature immune systems [16]. Previous studies have linked age under five years and underlying diseases to severe illness or death in children with influenza [17,18]. This study, however, did not identify age or underlying diseases as independent predictors for mortality among critically ill children with influenza. Receiving more attention from parents and clinicians, young children and those with underlying diseases tend to obtain earlier and more aggressive medical interventions, potentially altering the natural disease course and lowering mortality risk. Moreover, studies have revealed an increasing age trend among children developing severe illness from influenza virus infection in the post-pandemic era [19,20]. Clinicians should recognize this potential epidemiological shift, maintaining high vigilance when assessing school-age children.

Confusion showed the most significant difference between the two groups among clinical manifestations (55.9% vs. 10.1%, *p* < 0.001). Altered consciousness status, a core indicator of critical illness, may result either from direct pathogen invasion of the central nervous system or from cerebral perfusion disorders secondary to severe infection or shock [21]. Consistent with previous findings on severe influenza, this observation further confirms the clinical importance of consciousness assessment [22]. Previous studies have reported bacterial pneumonia in over 20% of influenza patients [23,24], with those having bacterial co-infection facing higher risks of severe illness and death [25]. In this study, the non-survivor group showed higher complication rates across neurological, circulatory, respiratory, urinary, and digestive systems than the survivor group, suggesting a higher mortality risk for critically ill children with influenza complicated by multisystem damage [26]. Influenza-associated encephalopathy or encephalitis is a serious complication that may occur in children with influenza infection and may be associated with adverse clinical outcomes. The development and progression of severe influenza may involve multiple pathological processes, including excessive systemic inflammation, immune dysregulation, organ dysfunction, and neurological injury [6]. The findings of this study cannot be used to determine the risk of developing influenza-associated encephalopathy or encephalitis or to establish its specific prognostic value. Further studies are needed to clarify the relationship between influenza-associated neurological complications and mortality risk.

High serum urea, low albumin, and low platelet levels correlated significantly with mortality risk in critically ill children with influenza. UREA is a nitrogen-containing waste product of protein metabolism, primarily generated from ammonia through the hepatic urea cycle and subsequently excreted by the kidneys. Elevated serum UREA levels may indicate impaired renal excretion and can also be influenced by fluid status, protein catabolism, and disease severity. Therefore, UREA may reflect not only renal dysfunction but also the overall severity of illness in critically ill children [27]. Previous studies have shown that elevated UREA levels are associated with systemic inflammation and disease severity and may independently predict adverse clinical outcomes in patients with various infectious diseases, including infective endocarditis, Escherichia coli infection, and neonatal sepsis [28,29,30]. Serving as an important nutritional status marker, albumin also closely correlates with inflammatory response intensity. Lazar M et al. reported low albumin as an early prognostic factor for severe SARS-CoV-2 pneumonia outcomes, with mortality doubling for each 1.06 g/L decrease in serum albumin [31].

PLT, serving as a core mediator of hemostasis and thrombosis, also plays an important regulatory role in inflammation [32]. This study found that the platelet counts in children with influenza in the mortality group were significantly lower than those in the survival group; this may reflect severe pathological processes such as bone marrow suppression, immune exhaustion, or disseminated intravascular coagulation, consistent with previous studies [3,33]. In addition, some coagulation-related variables had a relatively high proportion of missing data in this study, with missing rates exceeding 10% for some variables. This was mainly because some coagulation tests were performed outside the hospital, and the original results could not be completely retrieved from the electronic medical records. Therefore, to reduce the potential impact of missing data on the stability of the analysis, coagulation-related variables were not included in the prediction model. This may limit the model’s ability to assess the risk of mortality associated with coagulation abnormalities. Future prospective multicenter studies should systematically collect coagulation-related variables and further evaluate their incremental predictive value.

This study has several limitations. First, based on a single center retrospective design with a relatively small sample size, the data may be subject to selection bias. Second, although internal validation used the Bootstrap method, it relied on the same dataset and could not fully avoid the risk of overfitting. In addition, as the model has only undergone development and internal validation, its stability and generalizability remain untested in prospective or external independent cohorts. Future multicenter prospective studies are needed to confirm whether the model can accurately predict mortality risk in children with severe influenza and to allow calibration or updating based on validation results.

In addition, the DCA results only reflect the potential net benefit of the model across different risk thresholds and should therefore be considered exploratory evidence. The nomogram may assist PICU clinicians with early mortality risk stratification after admission. All children in this study received antiviral treatment, which was generally initiated within 3 days of disease onset. However, this study did not systematically collect information on PICU severity scores, clinician risk assessment, or specific treatment decisions, precluding further evaluation of the incremental predictive value of the model compared with existing scoring systems, models based on baseline clinical variables, or clinical judgment. Prospective studies are needed to further validate its clinical utility. Finally, some treatment- and disease management-related information was incompletely collected, including influenza virus type, influenza vaccination history, bacterial coinfection, mechanical ventilation, and specific treatment measures. Changes in treatment strategies during the COVID-19 pandemic were also not systematically recorded. These factors may have affected the study findings to some extent, and future studies should incorporate relevant clinical and treatment information for further validation.

## 5. Conclusions

This study developed a nomogram model based on PLT, UREA, ALB, Hgb, and confusion to predict mortality risk in critically ill children with influenza. The model showed good discrimination and calibration in internal validation and demonstrated potential clinical net benefit. However, as this was a single-center retrospective study with internal validation only, the model should currently be considered an exploratory prediction tool. Its clinical utility and generalizability require further confirmation through large-scale multicenter studies and independent external validation.

## Figures and Tables

**Figure 1:**
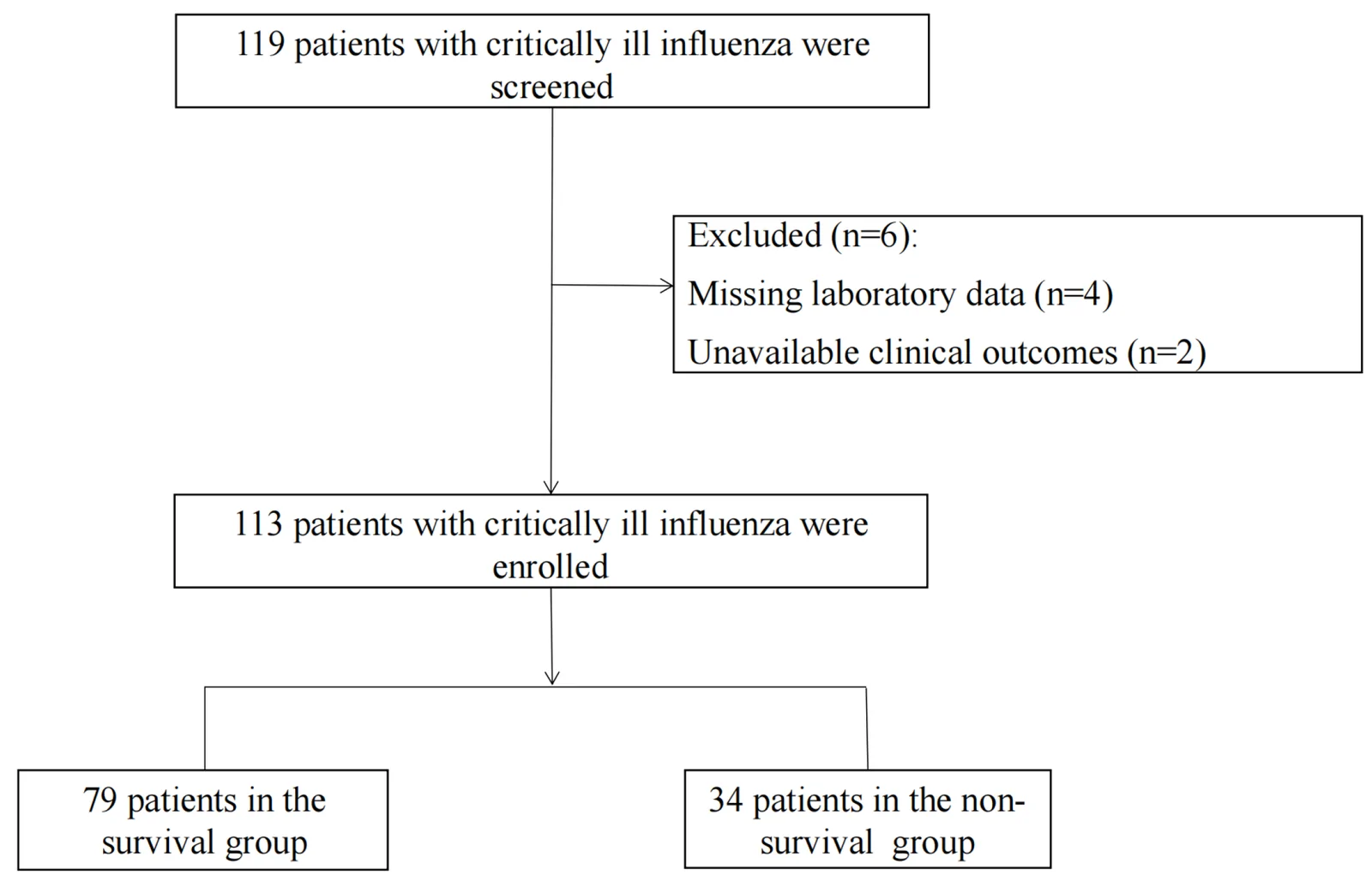
Flow chart of patient recruitment.

**Figure 2:**
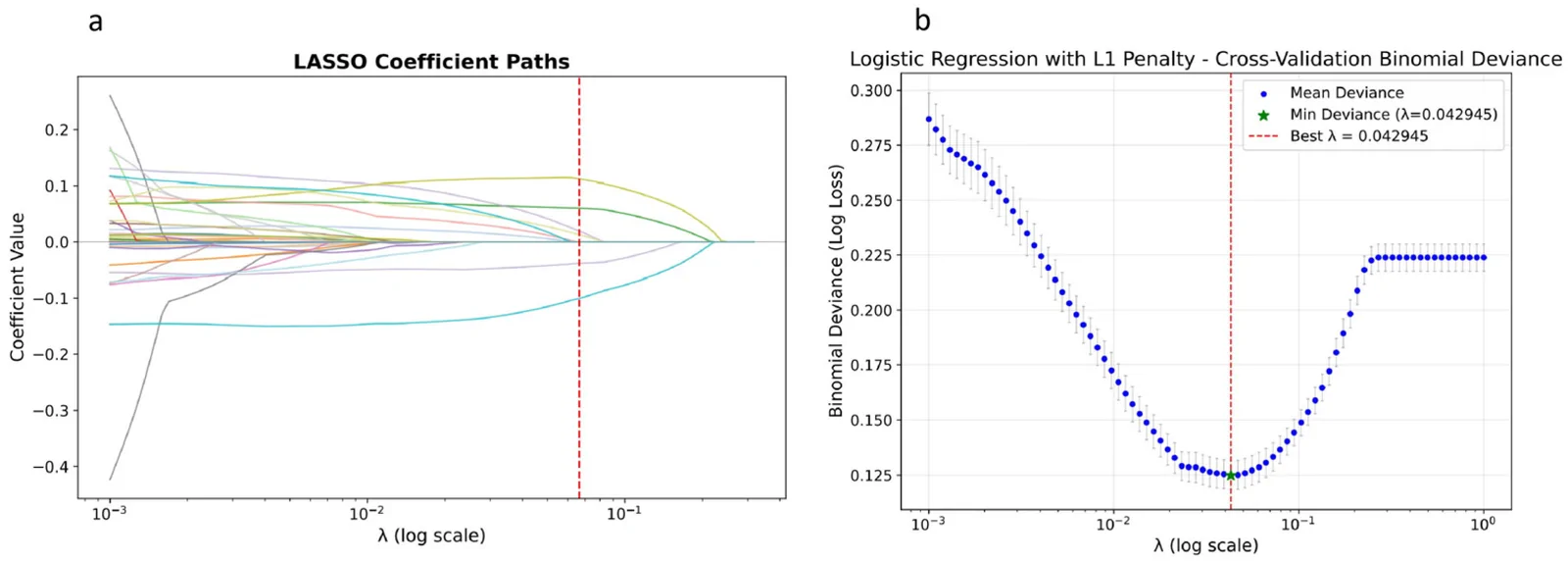
LASSO Logistic regression for variable selection. (**a**). LASSO coefficient path plot; (**b**). five-fold cross-validation for determining the optimal regularization parameter λ. The red dashed line indicates the optimal λ (λ = 0.042945).

**Figure 3:**
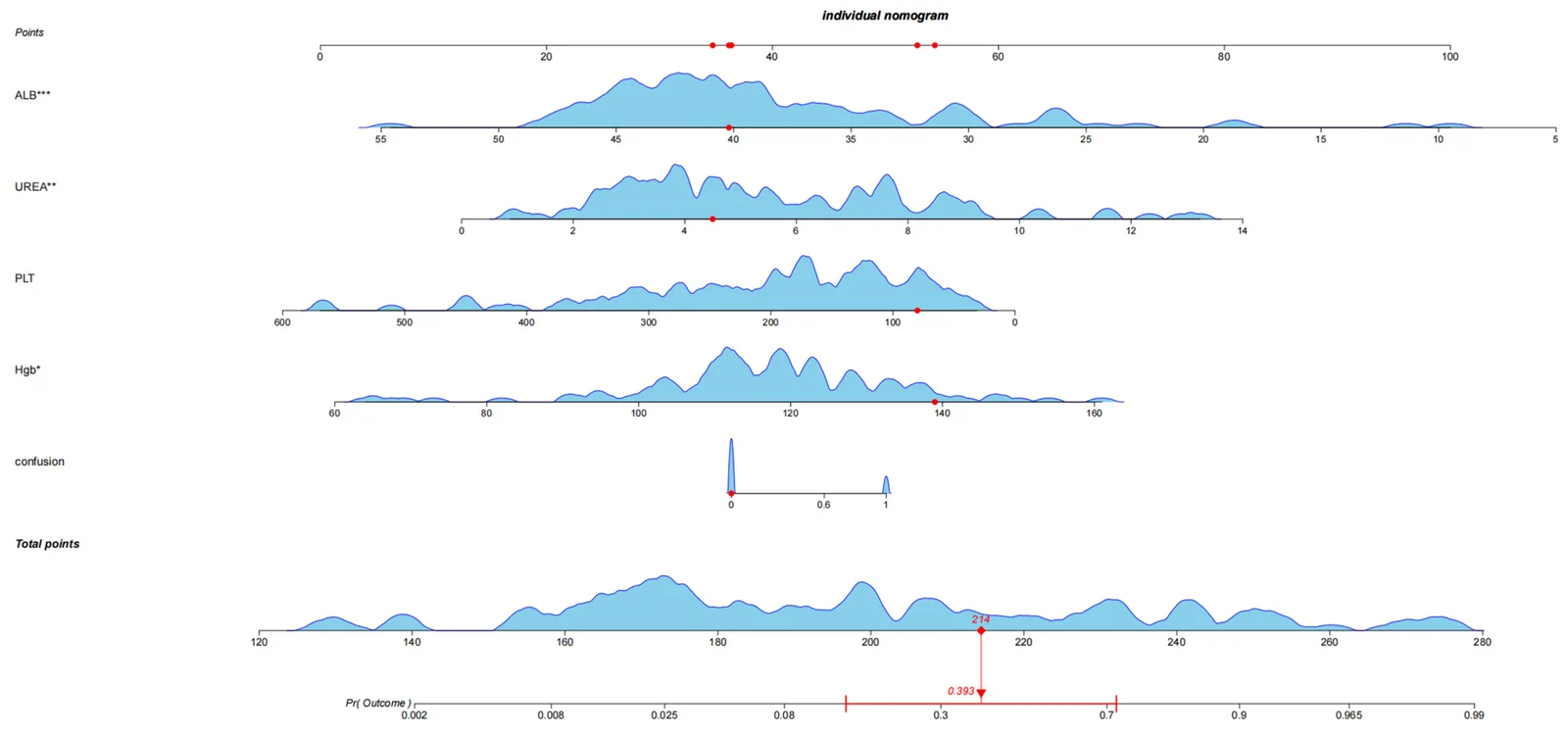
Nomogram for Predicting Mortality Risk in Critically Ill Children with Influenza. Note: * *p* < 0.05, ** *p* < 0.01, and *** *p* < 0.001. Abbreviations: ALB, albumin; PIL, platelet count; Hgb, hemoglobin.

**Figure 4:**
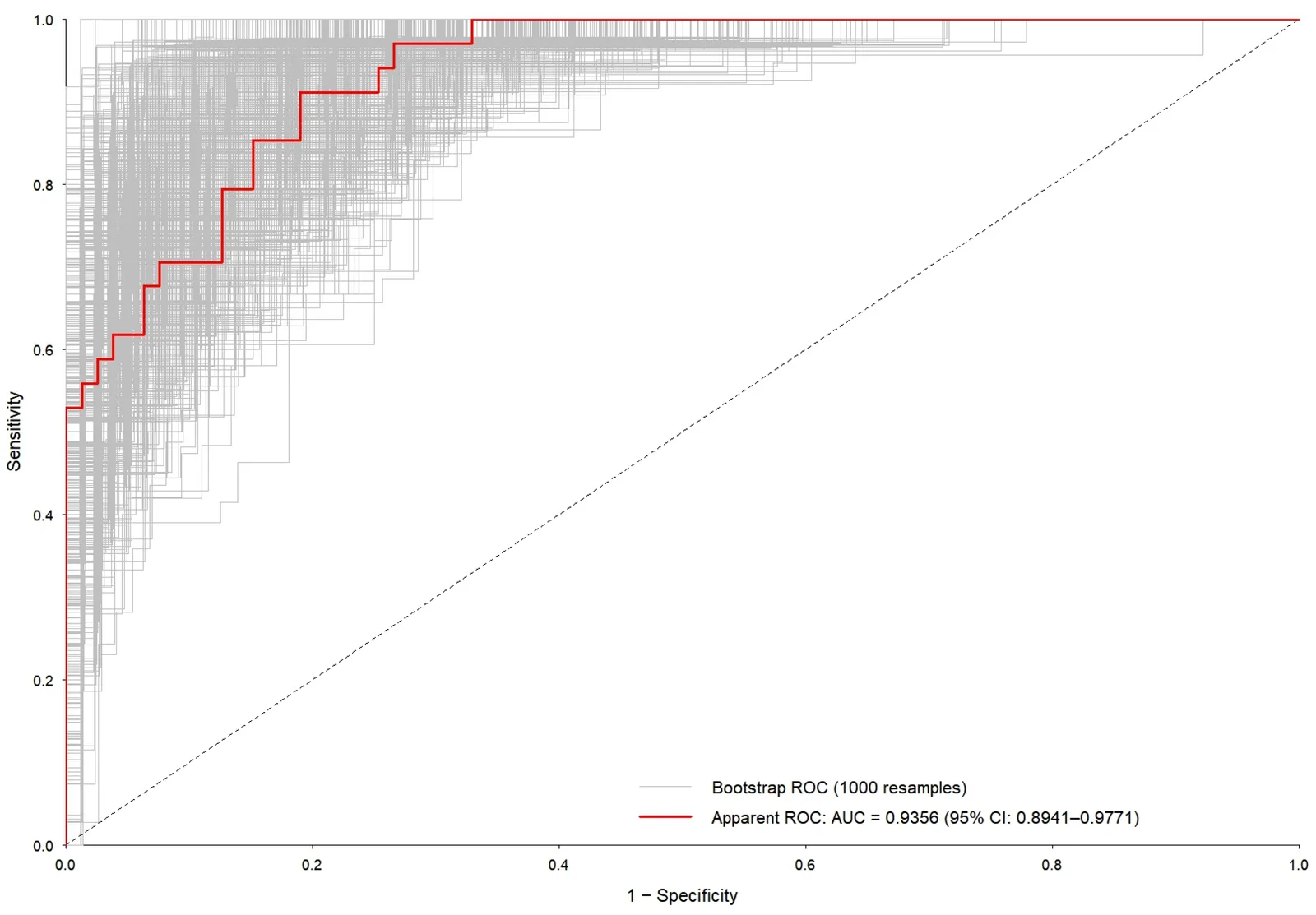
ROC Curve and Bootstrap Internal Validation of the Nomogram Model. Abbreviations: ROC, receiver operating characteristic; AUC, area under the curve; CI, confidence interval.

**Figure 5:**
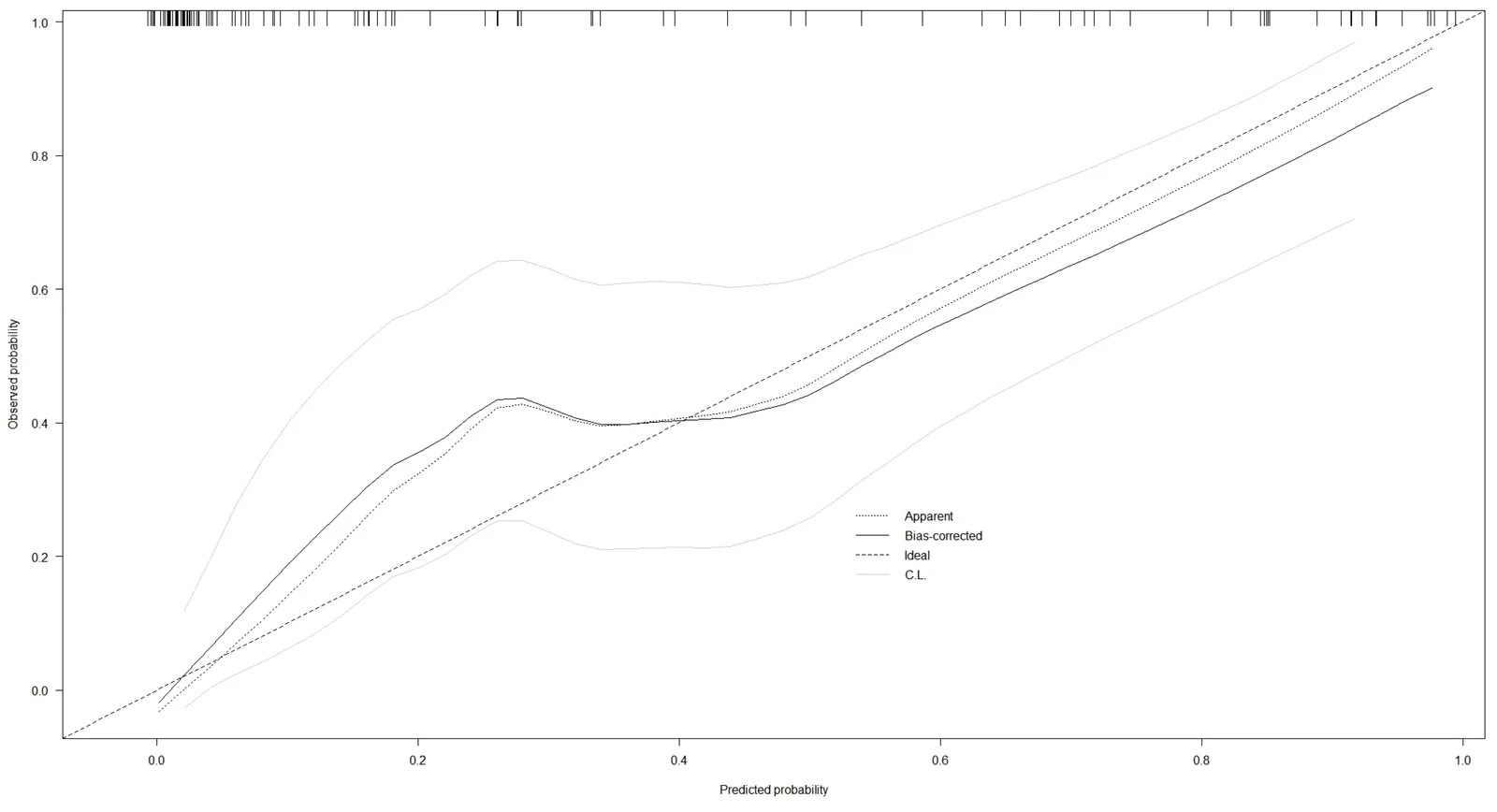
Calibration Curve of the Nomogram Model. Note: The calibration curve evaluates the agreement between the predicted and observed probabilities of mortality. The *x*-axis represents the predicted probability, and the *y*-axis represents the observed probability. The dashed line represents the ideal calibration line, the dotted line represents the apparent calibration curve, the solid line represents the bootstrap-corrected calibration curve, and the light gray curves represent the confidence limits of the calibration curve. The bootstrap-corrected calibration curve remained generally close to the ideal calibration line, indicating good calibration of the model.

**Figure 6:**
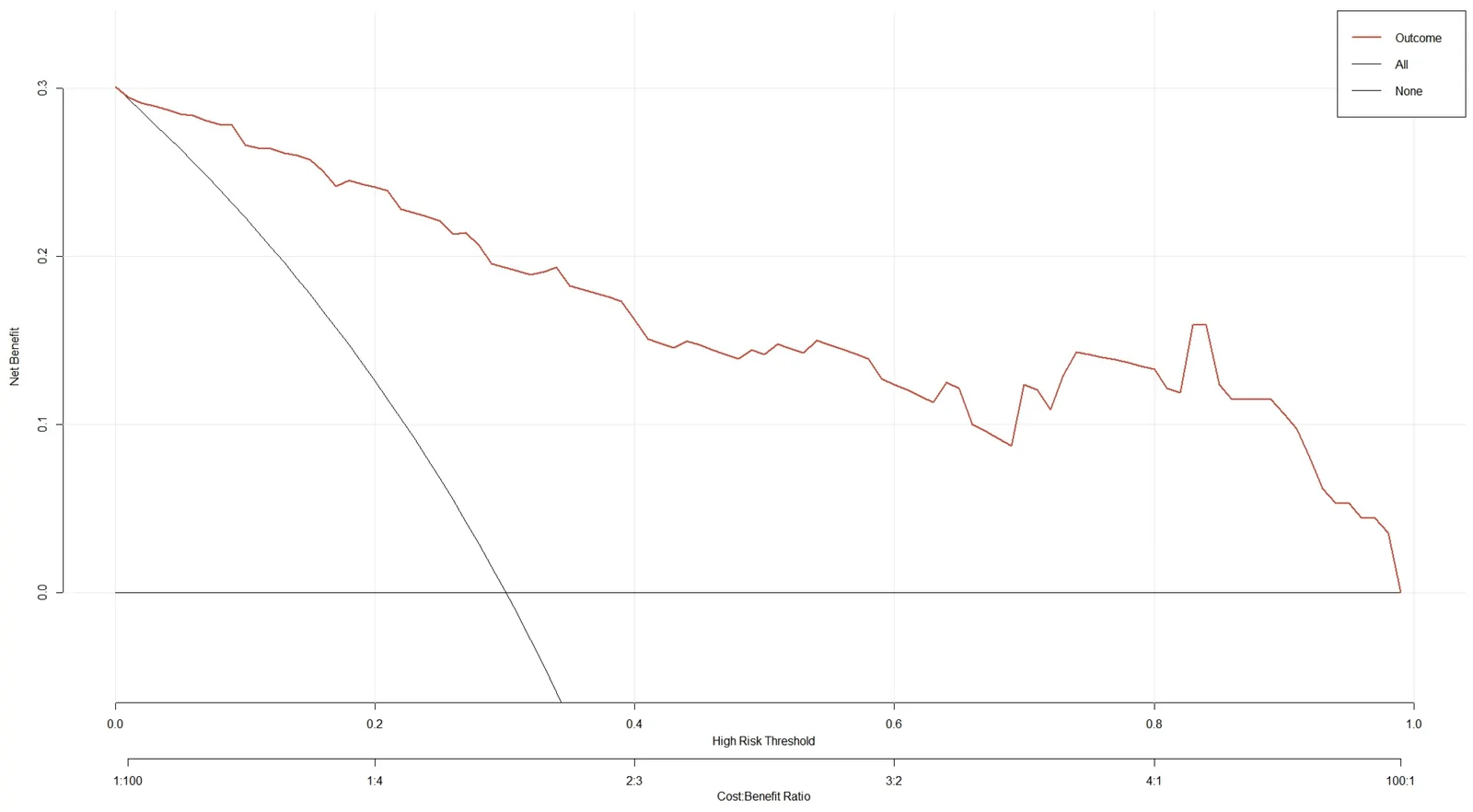
Decision Curve Analysis of the Nomogram Model. Note: Decision curve analysis was performed to evaluate the clinical net benefit of the nomogram model across different high-risk threshold probabilities. The *x*-axis represents the high-risk threshold probability, and the *y*-axis represents the net benefit. The red curve represents the nomogram model, the black solid line labeled “All” represents the strategy of intervening in all patients, and the black horizontal line labeled “None” represents the strategy of intervening in no patients. The model curve remained above the “All” and “None” strategies across a broad range of threshold probabilities, suggesting a potential clinical net benefit of the nomogram model. Abbreviation: DCA, decision curve analysis.

**Table 1:** Baseline characteristics between survivors and non-survivors.

Variables	Survival Group (*n* = 79)	Non-Survival Group (*n* = 34)	*p* Value
sex, *n* (%)			0.697
Male	41 (51.9)	19 (55.9)	
Female	38 (48.1)	15 (44.1)	
Age, years	5.50 (1.83, 7.25)	3.83 (2.50, 7.50)	0.873
Underlying disease, *n* (%)	26 (32.9)	10 (29.4)	0.714
Preterm birth, *n* (%)	6 (7.6)	1 (3.0)	0.347

Note: Values are *n* (%), median (interquartile range), unless otherwise noted. Statistical significance was defined as *p* < 0.05.

**Table 2:** Variable analysis using Firth penalized logistic regression.

Variables	β	OR	95% CI	*p* Value
PLT, 10^9^/L	−0.007668	0.992	0.984–1.000	0.0557
UREA, mmol/L	0.344343	1.411	1.131–1.872	0.0018
ALB, g/L	−0.142271	0.867	0.785–0.938	0.0004
Hgb, g/L	0.045652	1.047	1.010–1.093	0.0119
confusion	0.986438	2.682	0.746–9.787	0.1292

Note: Statistical significance was defined as *p* < 0.05. Abbreviations: PLT, platelets; ALB, albumin; Hgb, hemoglobin.

## Data Availability

The datasets used and/or analyzed during the current study are available from the corresponding authors on reasonable request.

## Supplementary Materials

The following supporting information can be downloaded at: https://www.mdpi.com/article/10.3390/children13101293/s1, Table S1: Comparison of clinical symptoms between survivors and non-survivors; Table S2: Comparison of Biomarkers between survivors and non-survivors; Table S3: Comparison of complications between survivors and non-survivors. Figure S1: Distributions of selected continuous laboratory variables in survivors and non-survivors.

## Abbreviations

The following abbreviations are used in this manuscript:

LASSO	Least absolute shrinkage and selection operator
ROC	Receiver operating characteristic
C-index	Concordance index
DCA	Decision curve analysis
PLT	Platelet
SIRI	Systemic inflammatory response index
ANE	Acute necrotizing encephalopathy
NLR	Neutrophil-to-lymphocyte ratio
PLR	Platelet-to-lymphocyte ratio
MLR	Monocyte-to-lymphocyte ratio
SII	Systemic immune inflammation index
WBC	White blood cells
NE	Neutrophil
CRP	C-reactive protein
TG	Triglycerides
TC	Total cholesterol
AKI	Acute kidney injury
CKD	Chronic kidney disease
Hgb	Hemoglobin

## References

[1] Nayak J., Hoy G., Gordon A. (2021). Influenza in Children. Cold Spring Harb. Perspect. Med..

[2] World Health Organization Influenza (Seasonal). https://www.who.int/en/news-room/fact-sheets/detail/influenza-(seasonal).

[3] Ozsurekci Y., Aykac K., Bal F., Bayhan C., Basaranoglu S.T., Alp A., Cengiz A.B., Kara A., Ceyhan M. (2021). Outcome predictors of influenza for hospitalization and mortality in children. J. Med. Virol..

[4] Tuckerman J., Misan S., Crawford N.W., Marshall H.S. (2019). Influenza in Children With Special Risk Medical Conditions: A Systematic Review and Meta-analysis. Pediatr. Infect. Dis. J..

[5] White E.B., O’Halloran A., Sundaresan D., Gilmer M., Threlkel R., Colón A., Tastad K., Chai S.J., Alden N.B., Yousey-Hindes K. (2023). High Influenza Incidence and Disease Severity Among Children and Adolescents Aged <18 Years—United States, 2022–23 Season. MMWR Morb. Mortal. Wkly. Rep..

[6] Sakuma H., Thomas T., Debinski C., Eyre M., Han V.X., Jones H.F., Kawano G., Lee V.W., Malone S., Matsuishi T. (2025). International consensus definitions for infection-triggered encephalopathy syndromes. Dev. Med. Child Neurol..

[7] Yang M., Yi L., Jia F., Zeng X., Liu Z. (2024). Characteristics and outcome of influenza-associated encephalopathy/encephalitis among children in China. Clinics.

[8] Fazal A., Harker E.J., Neelam V., Olson S.M., Rolfes M.A., Reinhart K., Kniss K., Frutos A., Leonard J., Reed C. (2025). Pediatric Influenza-Associated Encephalopathy and Acute Necrotizing Encephalopathy—United States, 2024–2025 Influenza Season. MMWR Morb. Mortal. Wkly. Rep..

[9] Minozzi S., Lytras T., Gianola S., Gonzalez-Lorenzo M., Castellini G., Galli C., Cereda D., Bonovas S., Pariani E., Moja L. (2022). Comparative efficacy and safety of vaccines to prevent seasonal influenza: A systematic review and network meta-analysis. EClinicalMedicine.

[10] Grohskopf L.A., Ferdinands J.M., Blanton L.H., Broder K.R., Loehr J. (2024). Prevention and Control of Seasonal Influenza with Vaccines: Recommendations of the Advisory Committee on Immunization Practices—United States, 2024–2025 Influenza Season. MMWR Recomm. Rep..

[11] Zhao L., Wang J., Xu J., Guo J., Zhang P., Guo X., Zuo Z., Gao R., Gao L., Wang J. (2026). Surveillance reveals a prevalent pediatric A(H1N1)pdm09 virus with hemagglutinin substitutions S137P-R142K-V152I that diminish vaccine efficacy. Virus Genes.

[12] Han K., Hou Z., Tu S., Liu M., Chantler T., Larson H. (2024). Factors Influencing Childhood Influenza Vaccination: A Systematic Review. Vaccines.

[13] Tsang T.K., Wang C., Tsang N.N.Y., Fang V.J., Perera R.A.P.M., Malik Peiris J.S., Leung G.M., Cowling B.J., Ip D.K.M. (2023). Impact of host genetic polymorphisms on response to inactivated influenza vaccine in children. npj Vaccines.

[14] China Medicine Education Association Committee on Pediatrics, The Subspecialty Group of Respiratory Diseases, Society of Pediatrics, Chinese Medical Association, Pediatric Respiratory Working Committee, Chinese Association of Chest Physicians, Pediatric Committee of Chinese Research Hospital Association, Pediatric Committee of Chinese Non-Government Medical Institutions Association, Child Health and Drug Research Committee of China Association of Traditional Chinese Medicine, Children’s Safe Medication Branch of China Medical News and Information Association, Pediatric Expert Committee of China Primary Health Care Foundation, Global Pediatric Respiratory Alliance, Beijing Yaodun Public Welfare Foundation (2024). Guideline for diagnosis, treatment and prevention of influenza in children (medical version, 2024). Chin. J. Appl. Clin. Pediatr..

[15] Baran A.I., Binici I., Arslan Y., Hakseven Karaduman Z., Ilter S., Tarcan T., Unal M. (2024). Hematologic Inflammation Indices for Differentiating between Brucella, Pyogenic, and Tuberculous Spondylodiscitis. Biomedicines.

[16] Dondi A., Guida F., Trombetta L., De Peppo Cocco M., Piccirilli G., Andreozzi L., Battelli E., Castaldo P., Corsini I., Pierantoni L. (2025). Burden and Clinical Characteristics of Influenza and Its Complications in Children Across Multiple Epidemic Seasons. Viruses.

[17] Wei J., Huang H., Wu X., Xu Y., Wang X. (2026). Pathogenesis and Research Models of Acute Influenza-Associated Encephalitis/Encephalopathy: An Update. Viruses.

[18] Hayek H., Noble E.K., Stewart L.S., Sahni L.C., Boom J.A., Michaels M.G., Williams J.V., Englund J.A., Klein E.J., Staat M.A. (2026). Influenza Vaccine Effectiveness Among Children With and Without Underlying Conditions. Pediatrics.

[19] Jia W., Zhang X., Sun R., Li P., Zhen X., Li Y., Wang D., Li C., Song C. (2024). Changes in the epidemiological characteristics of influenza in children in Zhengzhou, China, in the post-COVID-19 era. BMC Public Health.

[20] Liu H.F., Hu X.Z., Liu C.Y., Guo Z.H., Lu R., Xiang M., Wang Y.-Y., Yin Z.-Q., Wang M., Sui M.-Z. (2024). Development and validation of a nomogram for predicting occurrence of severe case in children hospitalized with influenza A (H1N1) infection during the post-COVID-19 era. Heliyon.

[21] Kim J., Erice C., Rohlwink U.K., Tucker E.W. (2022). Infections in the Developing Brain: The Role of the Neuro-Immune Axis. Front Neurol..

[22] Silverman A., Walsh R., Santoro J.D., Thomas K., Ballinger E., Fisher K.S., Thomas A.X., Appavu B., Kruer M.C., Influenza-Associated Acute Necrotizing Encephalopathy (IA-ANE) Working Group (2025). Influenza-Associated Acute Necrotizing Encephalopathy in US Children. JAMA.

[23] Lei B., Wang S., Yu L., Ma Q. (2025). Post-influenza bacterial infection: Mechanisms of pathogenesis and advances in therapeutic strategies. Front Microbiol..

[24] Qiao M., Moyes G., Zhu F., Li Y., Wang X. (2023). The prevalence of influenza bacterial co-infection and its role in disease severity: A systematic review and meta-analysis. J. Glob. Health.

[25] Bartley P.S., Deshpande A., Yu P.C., Klompas M., Haessler S.D., Imrey P.B., Zilberberg M.D., Rothberg M.B. (2022). Bacterial coinfection in influenza pneumonia: Rates, pathogens, and outcomes. Infect. Control Hosp. Epidemiol..

[26] Sun R., Zhang X., Jia W., Li P., Song C. (2024). Analysis of clinical characteristics and risk factors for death due to severe influenza in children. Eur. J. Clin. Microbiol. Infect. Dis..

[27] Shen J., Wang Z., Liu Y., Wang T., Wang X.Y., Qu X.H., Chen Z.-P., Han X.-J. (2024). Association of blood urea nitrogen with all-cause and cardiovascular mortality in hyperlipidemia: NHANES 1999-2018. Lipids Health Dis..

[28] Wang Q., Qiu J., Huang J.L., Jiang M., Lu J.Q., Wu D., Wei X.-B., Yu D.-Q. (2023). Prognostic Value of Blood Urea Nitrogen for Short-Term Mortality in Patients with Infective Endocarditis. Infect. Dis. Ther..

[29] Zou X.L., Feng D.Y., Wu W.B., Yang H.L., Zhang T.T. (2021). Blood urea nitrogen to serum albumin ratio independently predicts 30-day mortality and severity in patients with *Escherichia coli* bacteraemia. Med. Clin..

[30] Li X., Li T., Wang J., Dong G., Zhang M., Xu Z., Hu Y., Xie B., Yang J., Wang Y. (2021). Higher blood urea nitrogen level is independently linked with the presence and severity of neonatal sepsis. Ann. Med..

[31] Lazar M., Barbu E.C., Chitu C.E., Anghel A.M., Niculae C.M., Manea E.D., Damalan A.C., Bel A.A., Patrascu R.E., Hristea A. (2022). Mortality Predictors in Severe SARS-CoV-2 Infection. Medicina.

[32] Mandel J., Casari M., Stepanyan M., Martyanov A., Deppermann C. (2022). Beyond Hemostasis: Platelet Innate Immune Interactions and Thromboinflammation. Int. J. Mol. Sci..

[33] Yardımcı A.C., Yıldız S., Ergen E., Ballı H., Ergene E., Guner Y.S., Karnap M., Keskin D.D., Yuksel H., Bocutoglu F. (2021). Association between platelet indices and the severity of the disease and mortality in patients with COVID-19. Eur. Rev. Med. Pharmacol. Sci..

