# Supplementary material for: Development of a Nomogram Model for Predicting Mortality Risk in Critically Ill Children with Influenza
Source: Children (Basel). 2026 Sep 23;13(10):1293. doi: 10.3390/children13101293 (PMC13703509; doi:10.3390/children13101293)
Supplement: Supplementary file 1 [file children-13-01293-s001.zip › Supplementary Material 1--Table S1.pdf]

Table S1. Comparison of clinical symptoms between survivors and non-survivors.

Variables	Survival group（n=79）	Non-survival group（n=34）	P value
Fever days, days	4.00（3.00,7.00）	2.00（2.00,4.00）	0.001
Cough, n (%)	55（69.6）	28（82.4）	0.160
Rhinorrhoea, n (%)	21（26.6）	9（26.5）	0.990
Emesis, n (%)	35（44.3）	21（61.8）	0.089
Diarrhoea, n (%)	11（13.9）	6（17.6）	0.612
Anorexia, n (%)	41（51.9）	15（44.1）	0.448
Seizure, n (%)	46（58.2）	22（64.7）	0.519
Dyspnea*, n (%)	22（27.8）	4（11.8）	0.062
Confusion, n (%)	8（10.1）	19（55.9）	＜0.001
Hospital stays, days	11.00（7.00,20.00）	3.00（2.00,10.00）	＜0.001

Note: Values are n (%), median (interquartile range), unless otherwise noted. Statistical significance was defined as $P < 0.05$. * Fisher's exact test; other categorical variables were compared using Pearson's chi-square test.
