# Supplementary material for: Development of a Nomogram Model for Predicting Mortality Risk in Critically Ill Children with Influenza
Source: Children (Basel). 2026 Sep 23;13(10):1293. doi: 10.3390/children13101293 (PMC13703509; doi:10.3390/children13101293)
Supplement: Supplementary file 1 [file children-13-01293-s001.zip › Supplementary Material 2--Table S2.pdf]

Table S2. Comparison of biomarkers between survivors and non-survivors.

Variables	Survival group (n=79)	Non-survival group (n=34)	P value
WBC, 10^9/L	7.91（5.86,12.01）	8.49（5.07,11.81）	0.726
NE%, %	82.7（64.9,91.1）	74.2（47.1,85.1）	0.021
LY%, %	11.8（5.9,23.9）	24.7（13.0,47.4）	0.004
NE, 10^9/L	6.05（3.88,10.41）	5.54（3.13,8.90）	0.499
LY, 10^9/L	0.88（0.53,1.50）	1.69（0.91,3.49）	0.002
MO%, %	5.20（2.30,7.60）	2.60（1.50,4.40）	＜0.001
MO, 10^9/L	0.38（0.19,0.74）	0.21（0.10,0.33）	0.001
Hgb, g/L	115.0（110.0,123.0）	123.0（108.0,136.0）	0.021
PLT, 10^9/L	204.0（159.0,300.0）	115.0（72.0,145.0）	＜0.001
CRP, mg/L	8.02（2.54,26.18）	10.12（3.29,18.25）	0.634
NLR	6.67（2.60,15.44）	2.92（1.06,6.55）	0.008
PLR	238.51（121.99,408.82）	81.62（27.15,171.05）	＜0.001
MLR	0.42（0.19,0.75）	0.13（0.04,0.27）	＜0.001
SII	1551.87（497.95,3674.48）	367.30（70.81,1140.59）	＜0.001
SIRI	2.35（0.60,6.01）	0.67（0.15,1.55）	＜0.001
CKMB, ng/mL	2.10（1.10,4.57）	2.90（1.70,8.37）	0.006
AST, U/L	39.0（31.8,65.6）	267.4（105.9,548.0）	＜0.001
ALT, U/L	18.8（14.0,44.0）	77.0（36.2,215.6）	＜0.001
GGT, U/L	11.9（9.0,23.6）	18.0（14.3,39.6）	0.001
LDH, U/L	326.0（272.5,445.8）	937.0（463.0,1396.0）	＜0.001
UREA, mmol/L	4.00（3.01,5.46）	7.53（6.22,8.58）	＜0.001
CREA, umol/L	31.4（22.8,43.0）	65.9（35.7,93.9）	＜0.001
UA, umol/L	274.0（213.2,394.8）	338.7（384.4,526.1）	＜0.001
TG, mmol/L	0.51（0.36,0.89）	0.79（0.33,1.86）	0.280
TC, mmol/L	3.42（2.94,4.07）	3.46（2.82,3.96）	0.803
Albumin, g/L	42.2（38.1,44.4）	35.8（27.9,39.5）	＜0.001
Globulin, g/L	22.7（19.9,25.5）	21.4（18.5,23.6）	0.244
A/G	1.83±0.41	1.57±0.39	＜0.001

Note: Values are n (%), mean±standard deviation or median (interquartile range), unless otherwise noted. Statistical significance was defined as $P < 0.05$. Abbreviations: WBC, white blood cells; NE, Neutrophil count; LY, Lymphocyte count; MO, Monocyte; Hgb, hemoglobin; PLT, platelets; CRP, C-reactive protein; NLR, Neutrophil-to-lymphocyte ratio; PLR, Platelet-to-lymphocyte ratio; MLR, monocyte-to-lymphocyte ratio; SII, systemic immune inflammation; SIRI, systemic inflammatory response index; CKMB, Creatine kinase isozyme; AST, Aspartate aminotransferase; ALT, Alanine aminotransferase; GGT, Glutamyl transpeptidase; LDH, Lactate dehydrogenase; CREA, Creatinine; UA, Uric Acid; TG, Triglyceride; TC, Total Cholesterol; A/G, Albumin-to-globulin ratio.
