# Supplementary material for: Development of a Nomogram Model for Predicting Mortality Risk in Critically Ill Children with Influenza
Source: Children (Basel). 2026 Sep 23;13(10):1293. doi: 10.3390/children13101293 (PMC13703509; doi:10.3390/children13101293)
Supplement: Supplementary file 1 [file children-13-01293-s001.zip › Supplementary Material 3--Figure S1.pdf]

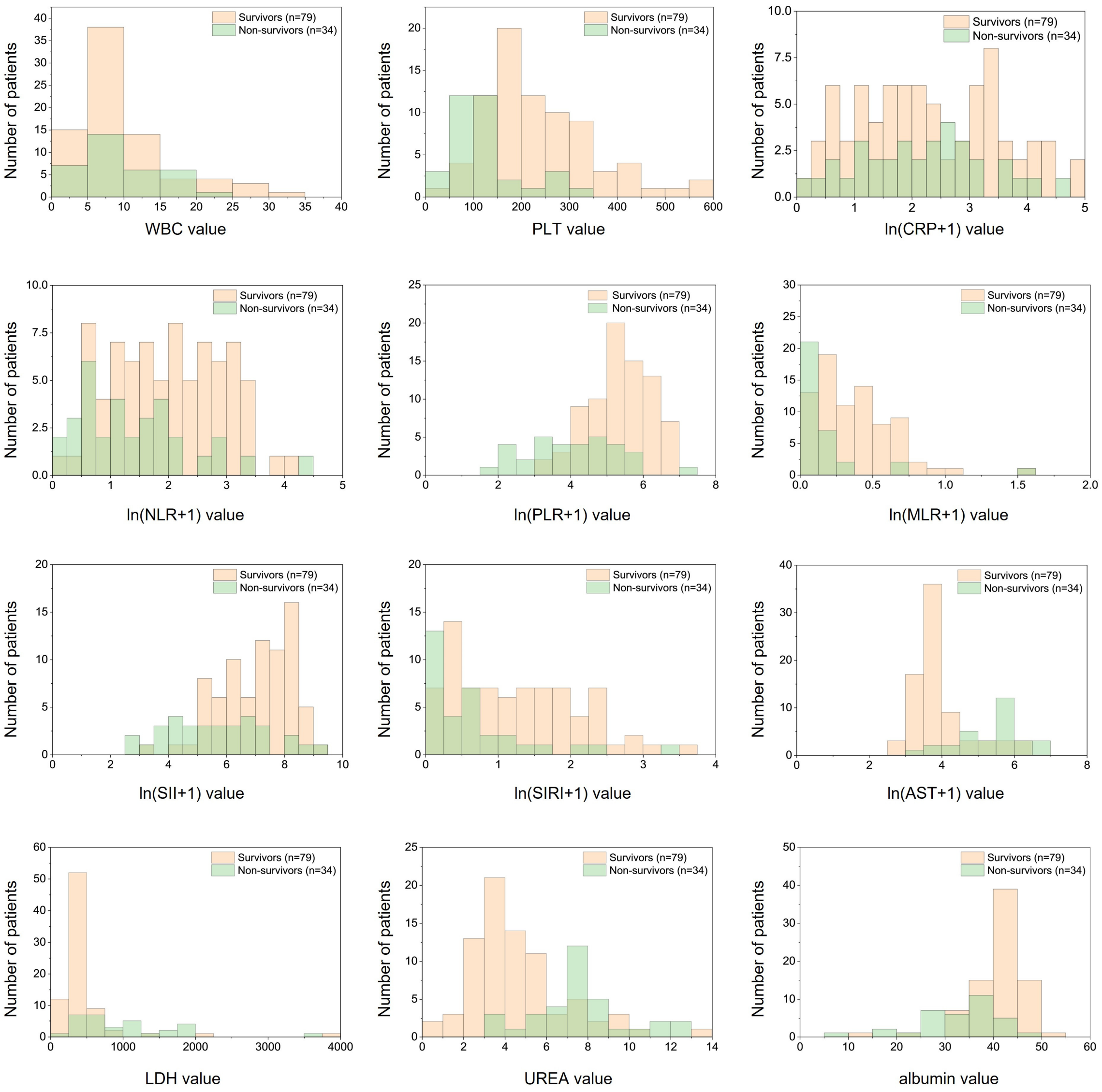

Figure S1. Distributions of selected continuous laboratory variables in survivors and non-survivors.

Note: Histograms compare survivors (n = 79) and non-survivors (n = 34). Natural logarithmic transformation after adding 1 was applied to markedly right-skewed variables, as indicated on the x-axis. Orange represents survivors, and green represents non-survivors.

Abbreviations: WBC, white blood cell count; PLT, platelet count; CRP, C-reactive protein; NLR, neutrophil-to-lymphocyte ratio; PLR, platelet-to-lymphocyte ratio; MLR, monocyte-to-lymphocyte ratio; SII, systemic immune-inflammation index; SIRI, systemic inflammatory response index; AST, aspartate aminotransferase; LDH, lactate dehydrogenase; UREA, urea; ALB, albumin.
