# Supplementary material for: Development of a Nomogram Model for Predicting Mortality Risk in Critically Ill Children with Influenza
Source: Children (Basel). 2026 Sep 23;13(10):1293. doi: 10.3390/children13101293 (PMC13703509; doi:10.3390/children13101293)
Supplement: Supplementary file 1 [file children-13-01293-s001.zip › Supplementary Material 4--Table S3.pdf]

Table S3. Comparison of complications between survivors and non-survivors.

Variables	Total （n=113）	Survival group （n=79）	Non-survival group （n=34）	P value
Influenza-associated encephalopathy or encephalitis, n (%)	51（45.1）	23（29.1）	28（82.4）	＜0.001
Severe pneumonia, n (%)	36（31.9）	26（32.9）	10（29.4）	0.714
Respiratory failure, n (%)	44（38.9）	18（22.8）	26（76.5）	＜0.001
Sepsis, n (%)	28（24.8）	8（10.1）	20（58.8）	＜0.001
Coagulation dysfunction, n (%)	27（23.9）	6（7.6）	21（61.8）	＜0.001
Heart failure, n (%)	29（25.7）	9（11.4）	20（58.8）	＜0.001
Kidney injury or renal insufficiency*, n (%)	16（14.2）	3（3.8）	13（38.2）	＜0.001
Shock, n (%)	33（29.2）	8（10.1）	25（73.5）	＜0.001
Multiple organ dysfunction syndrome*, n (%)	20（17.7）	2（2.5）	18（52.9）	＜0.001
Gastrointestinal bleeding*, n (%)	11（9.7）	1（1.3）	10（29.4）	＜0.001

Note: Values are n (%), median (interquartile range), unless otherwise noted. Statistical significance was defined as $P < 0.05$.* Fisher's exact test; other categorical variables were compared using Pearson's chi-square test.
